# Reversal Training Discloses Gender Differences in a Spatial Memory Task in Humans

**DOI:** 10.3390/brainsci13050740

**Published:** 2023-04-29

**Authors:** Laura Tascón, Irene León, Rubén Fernández, José Manuel Cimadevilla

**Affiliations:** 1 Department of Psychology, University of Cordoba, C/San Alberto Magno, s/n, 14071 Cordoba, Spain; ltascon@uco.es; 2 Facultad de Educación, Universidad Internacional de La Rioja (UNIR), Av. de la Paz, 137, 26006 Logroño, Spain; irene.leon@unir.net; 3 Faculty of Health Sciences, University of Almeria, Carretera de Sacramento s/n, 04120 Almeria, Spain; rubenfer@ual.es; 4 Faculty of Psychology, University of Almeria, Carretera de Sacramento s/n, 04120 Almeria, Spain; 5 Health Research Center, University of Almeria, Carretera de Sacramento s/n, 04120 Almeria, Spain

**Keywords:** flexibility, sex differences, dimorphism, virtual reality, hippocampus

## Abstract

Over the past few years, spatial memory has been studied using virtual-reality-based tasks. Reversal learning has been widely used in spatial orientation tasks for testing, among other things, new learning and flexibility. By means of a reversal-learning protocol, we assessed spatial memory in men and women. A total of sixty participants (half of them women) performed a task that included two phases: during the acquisition phase, participants were asked to find one or three rewarded positions in the virtual room across ten trials. During the reversal phase, the rewarded boxes were moved to a new position and maintained for four trials. The results showed that men and women differed in the reversal phase, with men outperforming women in high demanding conditions. Dissimilarities in several cognitive abilities between both genders are the base of these differences and are discussed.

## 1. Introduction

Spatial memory involves encoding information about the environment, the landmarks within it, the layouts and the spatial goals we want to achieve. All of this information is stored in our memory to be retrieved in recall tasks [1]. The proper functioning of spatial memory depends on the integrity of the hippocampus and other brain structures [2,3,4]. In many cases, the relocation of objects to new positions challenges our mental flexibility and requires modifying our actions according to the environmental feedback.

Mental flexibility is important for facing a multitude of cognitive tasks in our daily life. This ability allows us to readjust our behavior when our performance does not reach the expected results in different social, emotional and cognitive contexts, which remain in continuous change [5]. 

Neuropsychological tests are typically used to evaluate cognitive flexibility. The gold standard is the Wisconsin Card Classification Test [6], which analyzes the ability to assume a rule change in a card classification task. There are also batteries composed of a list of questions that analyze the adaptability in different situations of everyday life. These include the Cognitive Flexibility Scale [7] and the Cognitive Flexibility Inventory [8]. 

Nevertheless, cognitive flexibility can also be evaluated through reversal learning tasks. In accordance with Colman [9], reversal learning is a form of learning in which an organism choosing repeatedly between two alternatives, A and B, is initially rewarded for choosing alternative A rather than B until a behavioral preference for A is established, and is then rewarded for choosing B rather than A. 

Spatial reversal learning has been frequently used in rodent research [10,11,12,13,14]. It consists of changing a spatial target to a new position, demanding subjects to extinguish previous spatial information in order to encode a new relationship between the target and the cues available. A correct performance in this type of task demands a good interaction of the prefrontal cortex and the hippocampal system [15,16]. In humans, reversal tasks have been highly studied [17,18], but few of them have used a spatial context [19] and allocentric tasks [20], which are hippocampal-dependent [21,22,23].

Moreover, the performance of both sexes in spatial memory tasks has been deeply studied and it is well-known that this cognitive ability is dimorphic [20,24,25,26,27,28,29,30,31]. On one hand, men usually use allocentric strategies, which are independent of the point of view [32,33]. This allows them to create flexible relationships between the landmarks available in the environment and the targets. In addition, men have a greater ability to create cognitive maps and show better performances in mental rotation tasks compared to women [34,35]. Similarly, men are prone to use Euclidean, geometric and distance information to orient themselves [36]. All of these skills are advantageous for performing allocentric tasks. On the other hand, women prefer to rely on self-centered reference frames based on detailed information, such as available landmarks [37,38].

It is important to point out that task difficulty could determine the appearance of sexual dimorphism, which could depend on the visuospatial working memory load [39,40]. Specifically, in the Boxes Room Task (described below), sexual differences are shown only at a specific level of difficulty [41]. At an intermediate level of difficulty, men outperform women [41]. This result is explained by the fact that men show a greater capacity for visuospatial working memory [42]. Accordingly, it is very important to adapt the difficulty level to the sample studied. 

In this study, men and women were compared in a reversal learning protocol in the Boxes Room Task. The aim of this work was to determine whether sex could influence reversal learning once the participants mastered the task. The Boxes Room Task [41] is a spatial memory test based on virtual reality technologies. It was shown to be useful for disclosing sex differences throughout the life cycle [28,30,41,43]. In addition, the level of difficulty can be easily modified, avoiding floor and ceiling effects. The performance of this task depends on the integrity of the medial temporal lobe [44]. We hypothesized that reversal learning would be more challenging for women in the more demanding test conditions. 

## 2. Materials and Methods

### 2.1. Participants

Thirty men and thirty women from the University of Almeria participated in this study (see Table 1). The participants were divided into two groups according to the level of difficulty of the experimental conditions. In the low difficulty protocol, the participants had to find one rewarded box, whereas in the intermediate difficulty protocol, the subjects had to find three rewarded boxes (Table 1).

None of the participants had any neurological disorder, psychological illness, intellectual disability or were on a drug treatment that could have affected their cognitive performance. This information was obtained through self-report. All participants were volunteers and they all agreed to sign the informed consent.

The study was conducted in accordance with the European Communities Council Directive 2001/20/EC and the Helsinki Declaration for biomedical research involving humans.

### 2.2. Apparatus

The Boxes Room Task was administered on a Hewlett Packard 2600-MHz notebook with a 15.4 TFT and a color screen (1920 × 1200 pixels). A Logitech joystick was used for the navigation through the virtual task. Visual and auditory feedbacks were emitted by the computer.

### 2.3. Procedure

The Boxes Room test is a virtual task in which 16 brown boxes were symmetrically distributed on a square and decorated room (see [41]). The room included several objects on the walls: the north wall was blank, the east wall had three Leonardo da Vinci paintings hanging, the south wall displayed some Egyptian ornaments and, finally, a door and a window were located on the west wall. The participants navigated using a joystick and selected the boxes by pressing one of their buttons. When the participants selected a box, two scenarios were possible: the box turned green (a rewarded box) or the box turned red (a non-rewarded box). The green boxes were also linked to a pleasant tune, which started when it was discovered, whereas for non-rewarded boxes, a rather discordant tune was heard. The individuals were asked to find those boxes that turned green when selected (rewarded boxes). The boxes remained green or red during the whole trial. The trial finished when all of the rewarded boxes were selected or when the elapsed time reached 150 s. When a new trial began, all of the boxes turned brown. 

The experimental session consisted of 14 trials, with an intertrial interval of five seconds. In the first 10 trials, the locations of the rewarded boxes remained constant; however, in trial 11, the rewarded boxes changed to a new position (reversal learning) and remained until the end of the experiment (trial 14). To avoid egocentric solutions, the starting point changed semi-randomly. There were two experimental difficulty conditions: low (one rewarded box) and medium (three rewarded boxes), and the participants were randomly assigned to them, keeping the same number of male and female participants in each group. 

As the intention was to facilitate both groups’ performance in the acquisition phase, three locations were established as the higher difficulty conditions. This level of difficulty was determined following [41], who found that men and women did not differ when searching for three rewards in the Boxes Room Task. In this way, men and women enjoyed the same learning opportunities before the reversal phase. No information regarding useful strategies, the location of the target boxes or any other features of the experiment was provided. The participants were not informed about the possibility of the boxes’ relocation. To avoid egocentric solutions, four different starting positions were used (north, south, east and west).

### 2.4. Statistical Analyses

The accuracy (by number of errors) and latencies (in seconds) during the initial 10 trials were analyzed through ANOVA (Gender × Trial) with repeated measures in the last variable. An analysis of trials 10 to 14 was carried out using the same statistical procedure. A Newman-Keuls test was applied for post-hoc analyses. STATISTICA version 8.0 was used. Differences were considered statistically significant for *p* < 0.05. 

## 3. Results

### 3.1. One Rewarded Box

**Figure 1:**
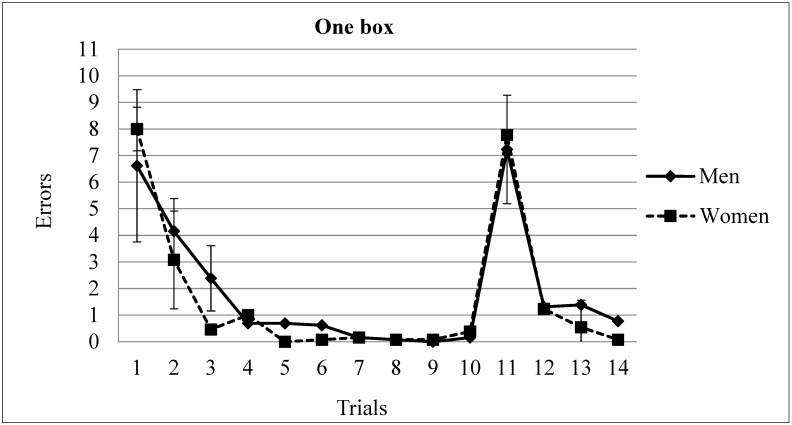
Number of errors committed by men and women in the one reward condition. The asymptotic level was reached in the 3rd trial. The location of the rewarded box was changed in trial 11. Participants reached the asymptotic in trial 12. Mean ± SEM.

Errors. Acquisition (Trials 1 to 10). A two-way ANOVA (Gender × Trial, with repeated measures in the last variable) revealed significant differences in factor Trial F(9,216) = 23.8, *p* = 0.0001. No differences were found in factor Gender F(1,24) = 0.61, *p* = 0.44 or Gender × Trial interaction F(9,216) = 0.87, *p* = 0.545. The post-hoc analysis of the trial factor revealed that the asymptotic level was reached in trial 3 (*p* < 0.05). The participants made more errors in trials 1 and 2 in comparison with the rest of the trials (see Figure 1).

Finally, an analysis of trials 10, 11, 12, 13 and 14 was run. An ANOVA (Gender × Trial, with repeated measures in the last variable) demonstrated significant differences in Trial F(4,96) = 55.95, *p* = 0.000, but no significant differences were found in Gender F(1,24) = 0.09, *p* = 0.76 nor in the Gender × Trial interaction F(4,96) = 0.52, *p* = 0.717 (see Figure 1). The post-hoc analysis of the trial factor revealed that the participants made more errors in trial 11, when the box location was moved to another position. There were no differences between trials 12 to 14 and trial 10. That is, after changing the box locations, the participants required only one trial to reach the asymptotic level of performance (mean of errors in trial 10 = 0.26; mean in trial 11 = 7.5; mean in trial 12 = 1.26; mean in trial 13 = 0.96; mean in trial 14 = 0.42) (see Figure 1).

**Figure 2:**
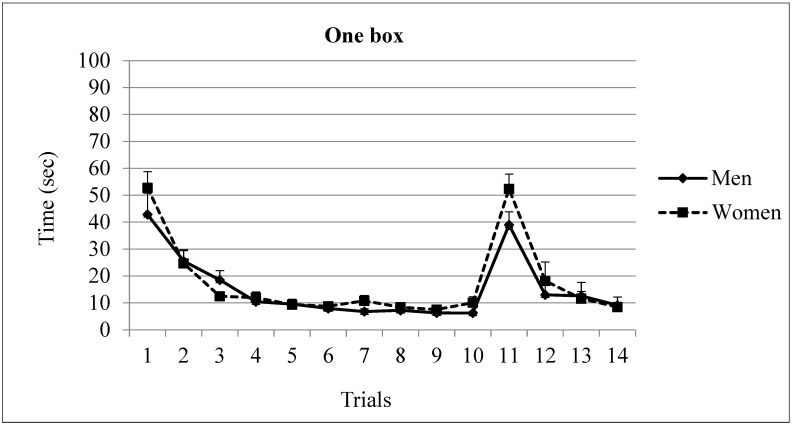
Time spent by men and women in one reward condition. The asymptotic level was reached in the 3rd trial. The location of the rewarded box was changed in trial 11. Afterwards, the asymptotic level was reached in trial 12. Mean ± SEM.

Latency. An ANOVA (Gender × Trial, with repeated measures in the last variable) of the latencies during trials 1 to 10 showed significant differences in Trial factor F(9,216) = 38.41, *p* = 0.0001 and no differences in Gender F(1,24) = 1.75, *p* = 0.197. There was not a significant main effect of the Gender × Trial interaction F(9,216) = 0.97, *p* = 0.463. The post-hoc analysis of trial factor revealed that the asymptotic level was reached in trial 3 (*p* < 0.001). Trials 1 and 2 showed differences with the rest of the trials (see Figure 2).

The analysis of trials 10 to 14 with a two-way ANOVA (Gender × Trial, with repeated measures in the last variable) revealed a significant main effect of Trial F(4,96) = 45.57, *p* = 0.000. There were no differences in Gender factor F(1,24) = 1.16, *p* = 0.291 or in the interaction term Gender × Trial F(2,96)= 1.57, *p* = 0.188 (see Figure 2). The post-hoc analysis of trial factor showed that after changing the target position, the asymptotic level was reached in trial 2. There were no significant differences between trial 10 and trials 12, 13 and 14 (mean of seconds in trial 10 = 8.15; mean in trial 11 = 45.69; mean in trial 12 = 15.57; mean in trial 13 = 12.11; and mean in trial 14 = 8.8) (see Figure 2).

### 3.2. Three Rewarded Boxes

**Figure 3:**
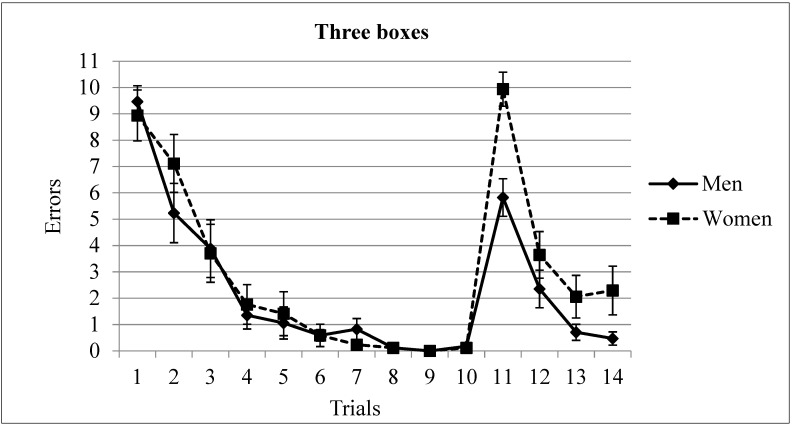
Number of errors committed by men and women in the three rewards condition. The asymptotic level was reached in trial 4. The location of the rewarded boxes was changed in trial 11, where men outperformed women. After changing the location, the asymptotic level was reached in the 3rd trial (trial 13). Mean ± SEM.

Errors. Acquisition (Trials 1 to 10). A two-way ANOVA (Gender × Trial, with repeated measures in the last variable) revealed significant differences in the factor Trial F(9,2889) = 59.99, *p* = 0.000, but not in Gender F(1,31) = 0.03, *p* = 0.775. The interaction Gender × Trial was not significant F(9,288) = 0.74, *p* = 0.669. The post-hoc analysis revealed that the asymptotic level was reached in trial 4 (*p* < 0.05) (see Figure 3).

A two-way ANOVA (Gender × Trial, with repeated measures in the last variable) of the number of errors on trials 10 to 14 demonstrated significant differences in the factors Trial F(4,128) = 65.44, *p* = 0.000 and Gender F(1,32) = 10.25 *p* = 0.003. The Gender × Trial interaction was also significant F(4,128) = 4.09, *p* = 0.003. The post-hoc analysis of Gender × Trial revealed that men committed less errors than women in trial 11 (mean of men = 5.82; mean of women = 9.94). In addition, both men and women reached the asymptotic level on trial 13; that is, in the third trial after changing the target position (see Figure 3). 

**Figure 4:**
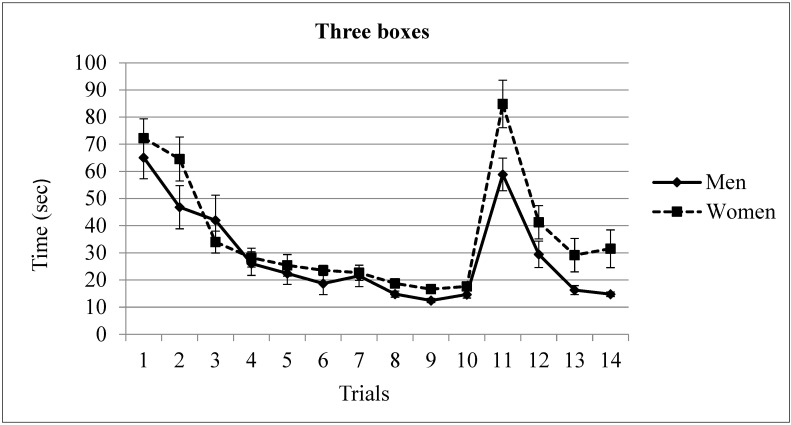
Time spent by men and women in the three rewards condition. The location of the rewarded boxes was moved in trial 11. Men spent shorter time than women after changing the target. Mean ± SEM.

Latency. An ANOVA (Gender × Trial, with repeated measures in the last variable) of the latencies in trials 1 to 10 revealed differences in Trial factor F(9,288) = 46.46, *p* = 0.0001. No significant differences emerged in Gender factor F(1,32) = 0.88, *p* = 0.353 or in the Gender × Trial interaction F(9,288) = 1.37, *p* = 0.197. The post-hoc analysis of trial factor confirmed that the asymptotic level was reached in trial 5 (*p* < 0.05). The participants spent more time on completing trials 1 to 4 than in the rest of the trials (see Figure 4). 

Finally, the analyses of the latencies in trials 10 to 14 revealed statistical differences in the factors Trial F(4,128) = 59.4, *p* = 0.000 and Gender F(1,32) = 8.44, *p* = 0.006. There was no significant main effect in the interaction term Gender × Trial F(4,128) = 2.06, *p* = 0.089. 

The post-hoc analysis of trial factor revealed that the participants spent more time on trial 11 (mean = 71.85 s) in comparison with the rest of the trials. In addition, in trial 10, the participants took less time (mean = 16.14) than in trials 12 (mean = 35.38 s) and 13 (mean = 22.73 s). Trial 14 (mean = 23.14 s) did not differ from trial 10 (mean = 16.14 s). Regarding Gender, men spent less time than women to complete the reversal task (mean of men = 26.81 s; mean of women = 40.89 s) (see Figure 4).

## 4. Discussion

This study shows that men and women differed in their performance during reversal training in a spatial memory task. Differences only appeared in the three boxes condition. The groups did not differ during acquisition, achieving a good level of performance in both conditions of difficulty. When searching for one rewarded position, they reached the asymptotic level in trial 3. A few more trials were needed to reach the asymptotic level when searching for three targets. However, men outperformed women during the reversal in the three boxes condition.

Our results are in line with other works in which dimorphism appeared between men and women. Since the pioneering work of Astur and colleagues using virtual reality techniques [24], many different studies have demonstrated that men and women differed in their spatial skills [20,21,40]. In all of these studies, men outperformed women in the virtual Morris water maze and virtual Walking Corsi test. In our study, differences emerged at a specific difficulty level. Usually, if demands are very low or high, both genders show a similar performance. However, the male participants outperformed their female counterparts at intermediate difficulty levels [29,41,43]. Specifically, the Boxes Room Task used by Canovas et al. [41] and Cánovas and Cimadevilla [45] demonstrated gender differences when five locations had to be remembered. In a similar spatial recognition task, Tascón García-Moreno and Cimadevilla [29] showed that men and women differed when two or three positions had to be remembered, but these differences disappeared when remembering one position. 

**Figure 5:**
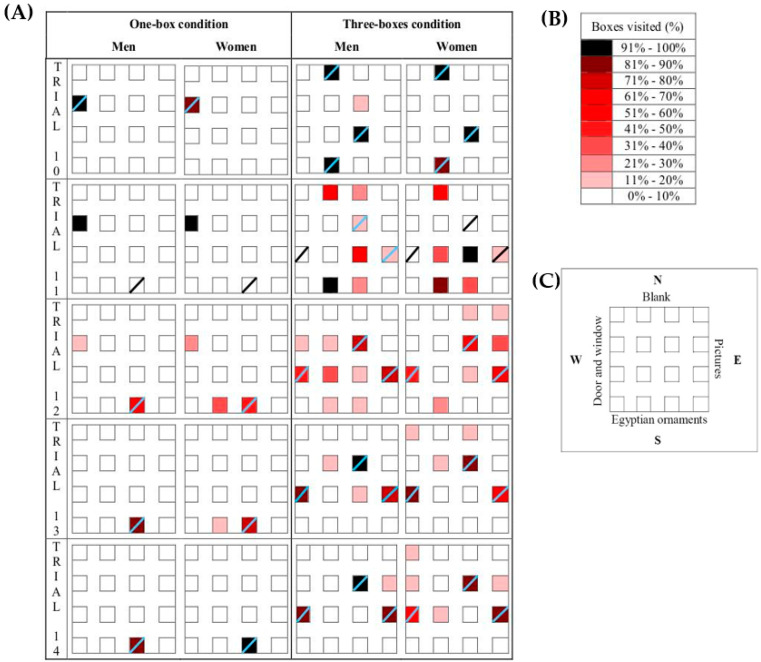
Zenithal view of the virtual room and the places selected by the participants in both difficulty conditions. (**A**) Positions selected by men and women in trials 10th to 14th. Boxes are colored according to the percentage of visits. For one reward condition only the first selected position is represented. For three rewards condition the first three selections are represented for each participant. Rewarded boxes were marked with an oblique line. (**B**) Caption about percentage of visits. (**C**) Schema about the room orientation and cues available.

In our study, the acquisition was similar in both groups and both conditions. Figure 5A represents the positions selected by men and women from trials 10 to 14. At both difficulty levels, in almost 100% of cases, the men and women selected the correct positions in trial 10, showing that they achieved good accuracy before the reversal phase. In trial 11, the rewarded boxes were moved to a new position. However, the participants visited the previous rewarded positions. Trials 12 to 14 showed the new learning. The reversal training demonstrated dimorphism in the three boxes condition as the women were slower than the men. It is possible that the women did not reach 91% of visits in any of the right positions. The women also committed more errors than the men in trial 11, although this performance is considered to be random as the new positions were moved to other locations. It is important to stress that reversal learning is very frequently used in spatial memory research as it increases the task demands. Participants not only have to extinguish memories about rewarding positions, but must also develop new memories that are incompatible with the previous ones. The reversal condition seems to be more discriminative than the initial learning in spatial tasks [20]. Thus, participants that mastered the initial training could be impaired in the reversal trials [20]. Our results are also in consonance with other studies that measured reversal learning in non-spatial tasks [18,46]. As in the current study, reversal modulates the task difficulty, highlighting differences between both sexes. 

Several explanations could account for this gender-specific behavior. Thus, dimorphism could be the result of participants possessing different levels of flexibility, with women persisting in previous targets. However, when compared graphically (see Figure 5), women did not visit previously rewarded positions more than men. In our test, women developed a tendency to visit a larger number of positions than men did, who, in turn, were more selective in their decisions. Thus, whereas in the three-reward condition, men chose a total of four different boxes in trial 14, women spread their search to seven different locations. This could explain the differences in the latency and could demonstrate that the spatial representation of the environment was more accurate in men. However, the women did not persist in visiting the previous goals. Therefore, gender differences cannot be explained in terms of cognitive flexibility. 

On the other hand, dimorphism could have emerged through differential abilities in imagery skills. Mental imagery is crucial to orientate through a space [47,48]. In each trial, the participants begin from a different wall in the room. This procedure avoids the use of egocentric solutions and forces the use of allocentric strategies. Accordingly, the participants needed to update the information about landmarks and location relationships. The abilities to form a cognitive map and to conduct mental rotation are helpful for achieving the task demands. Thus, differences could have emerged because men are better than women in mental rotation, as well as in composing cognitive maps [34,35], which help them to reorganize the information when the target locations are moved. However, as shown previously, both groups did not differ in the learning phase, and in this respect, this hypothesis is likely to have a minor role. Nevertheless, it should be borne in mind that these different abilities may have an impact on the chosen strategies when it comes to resolving the task. Regarding this, Goldberg [49] reported in the Cognitive Bias Task that men were prone to use dependence context strategies, whereas women preferred independent context strategies. Similarly, it was demonstrated that women usually use landmark or route strategies, whereas men chose survey strategies, which are related to metric and allocentric information and the capacity to create mental maps [40,50,51]. Geometrical information is more adequate than landmark information as information about distances and spatial relationships is essential for accurate performance in spatial orientation tasks [51]. This could be the reason why women had a tendency to be more disperse than men after the change of spatial location was produced (see Figure 5A). It is interesting to highlight that there were no landmarks on the north wall (see Figure 5C). Accordingly, the women could not rely on any cue for orientation and they made more mistakes when they opened the boxes that were close to this wall. 

Finally, another important factor that could contribute to the differences observed in this study is working memory load. Performance in this allocentric spatial memory task was reported to be conditioned by working memory capacity [52]. Wolbers and Hegarty [53] suggested the importance of working memory capacity to transform spatial cues into stable representations and keeping track of them. In our study, searching for three positions makes the task more complicated as the participants had to remember more locations. In addition, the reversal phase demanded a greater working memory load. The participants were not only required to maintain the previous locations in order to avoid them, but also the new ones to be selected. This could explain why dimorphism did not appear in the acquisition phase, but did it in the new learning, and only in the three boxes condition. This explanation is supported by other studies reporting sexual dimorphism in tasks with high visuospatial working memory load where participants had to elaborate, integrate and transform the visual imagined material. The differences decreased when the working memory load was reduced [31,41,54]. 

This study showed that the reversal protocol challenges spatial memory abilities, even in conditions accurately learned by participants. The dimorphism described cannot be explained as a lack of flexibility or inhibition, but is explained by other processes involved, such as better strategies being used by men or their higher spatial working memory capacity.

## Figures and Tables

**Table 1:** Distribution of participants between different task conditions.

Sample	Low Difficulty	High Difficulty
Men (n = 30)	13	17
Age	20.54 ± 2.96	21.59 ± 5.48
Women (n = 30)	13	17
Age	18.54 ± 0.88	20.76 ± 3.95

## Data Availability

Data available and can be received from the corresponding author under reasonable request.
